# Population dynamics of an RNA virus and its defective interfering particles in passage cultures

**DOI:** 10.1186/1743-422X-7-257

**Published:** 2010-09-29

**Authors:** Kristen A Stauffer Thompson, John Yin

**Affiliations:** 1 Department of Chemical and Biological Engineering, University of Wisconsin-Madison, Madison, Wisconsin 53706-1607 USA

## Abstract

**Background:**

Viruses can fall prey to their defective interfering (DI) particles. When viruses are cultured by serial passage on susceptible host cells, the presence of virus-like DI particles can cause virus populations to rise and fall, reflecting predator-prey interactions between DI and virus particles. The levels of virus and DI particles in each population passage can be determined experimentally by plaque and yield-reduction assays, respectively.

**Results:**

To better understand DI and virus particle interactions we measured vesicular stomatitis virus and DI particle production during serial-passage culture on BHK cells. When the multiplicity of infection (MOI, or ratio of infectious virus particles to cells) was fixed, virus yields followed a pattern of progressive decline, with higher MOI driving earlier and faster drops in virus level. These patterns of virus decline were consistent with predictions from a mathematical model based on single-passage behavior of cells co-infected with virus and DI particles. By contrast, the production of virus during fixed-volume passages exhibited irregular fluctuations that could not be described by either the steady-state or regular oscillatory dynamics of the model. However, these irregularities were, to a significant degree, reproduced when measured host-cell levels were incorporated into the model, revealing a high sensitivity of virus and DI particle populations to fluctuations in available cell resources.

**Conclusions:**

This study shows how the development of mathematical models, when guided by quantitative experiments, can provide new insight into the dynamic behavior of virus populations.

## Background

Defective interfering (DI) particles are virus-like byproducts of infections that are unable to infect cells on their own because they carry mutations, typically large deletions, in essential viral genes. However, when DI particles and standard virus particles co-infect the same cell the DI particles compete for resources that ultimately enable them to reproduce at the expense of virus particles. DI particles were discovered more than 60 years ago [1,2], and their generation by diverse DNA and RNA viruses has been widely documented [3-5], but it is not yet known what role DI particles may play in the behavior of natural infections. Defective viral genomes have been isolated from natural infections [5-8], but their potential for interference and their broader roles in viral pathogenesis remain open.

The unique biological properties of DI particles make them interesting and important for several reasons. First, their ability to interfere with standard virus infections highlights their potential use as therapeutic agents [9]. Moreover, the use of site-specific mutagenesis or reverse genetics to precisely create desired genomic defects, or introduce new functions, opens applications for DI particles as vaccines or prophylactics [10,11]. Second, their ability to activate innate immunity can alter the susceptibility of host cells to infection by standard virus [12,13]. Here, advances in the understanding of DI particle interactions with cells may suggest new ways to modulate how viruses grow and spread within a host. Third, mixtures of different DI particles can complement their own defects and thereby productively infect cells in the absence of standard virus [14-16]. This example provides an intriguing mechanism for viral infections to spread and persist in the absence of a single agent that can be isolated and cultured. Such mixtures may also offer advantages as a vaccines [17]. Finally, the ability of DI particles to adapt their degree of interference in response to mutation and selection of their co-infecting virus [18] may provide insights for the design of anti-viral strategies that resist escape [19,20].

A characteristic feature of DI particles is their emergence and outgrowth during high multiplicities of infection (MOI), where numerous copies of the standard virus infect each host cell. Under such conditions "predator" DI particles can productively interact with "prey" cells infected with standard virus. Ecological models of predator-prey behavior have been proposed to describe the dynamics of virus and DI particle populations [21-26], where the models have been loosely based on diverse measures of virus and DI particles, reviewed elsewhere [27]. However, the development of population dynamic models of virus and their DI particles have yet to be fully integrated with quantitative experiments. Recently, we used a yield-reduction assay to quantify how DI particles of vesicular stomatitis virus (VSV) impact the production of virus and DI particles in BHK cells, where we interpreted these measures using a mathematical model. The model enabled us to estimate parameters that describe how input levels of virus, DI particles and host cells interact to define outputs, specifically the production of virus and DI particles in a single passage [28]. Here we extend this data-driven modeling approach to better understand how virus and DI particle interactions impact their population behavior over multiple passages.

## Methods

### Cell and virus culture

The maintenance and analysis of host cell and virus cultures followed established methods [28]. Briefly, BHK-21 cells were cultured at 37°C and 5% CO_2 _in Eagle's Minimum Essential media (MEM, Cell Gro) with 5% GlutamaxTM (Gibco) with 10% fetal bovine serum (FBS, Hyclone); the media was switched 2% FBS for all virus infections. All studies employed VSV-N1, a molecularly well-defined virus strain based on the San Juan isolate of the Indiana serotype of VSV [29], generously provided by Gail Wertz. Virus stock was prepared by performing six plaque-to-plaque transfers, using 0.2 ml plaque-purified virus (10^4 ^PFU/ml) to inoculate 8 × 10^6 ^cells in a T-75 flask (BD Falcon), incubating 24 h at 37°C, passing through a 0.22 micron filter (Nalgene), and storing at -80°C. The stock titer was 1 × 10^9 ^PFU per ml, and the absence of detectable DI particle activity was confirmed by the yield-reduction assay.

### Yield-reduction assay for interference

The interference of VSV infection by DI particles was quantified by the yield-reduction assay [28,30]. Prior to all infections cells from cultures prepared in parallel were counted, enabling control of the multiplicity of infection (MOI), and quantification of virus yields and interference units on a per-cell basis. Cells were then infected with a range of serial dilutions, 1-to-10 or 1-to-2 of each sample, incubated 1 h at 37°C for adsorption, supplemented with stock virus to MOI 20, and incubated 1 h; then each culture well was rinsed twice with Hank's balanced salt solution (HBSS, Hyclone), replaced with MEM containing 2% FBS, incubated 24 h at 37°C to produce virus and DI particle progeny, and stored at -80°C until quantification by plaque assay.

### Serial-passage culture

Prior to each virus passage, fresh cells from parallel-prepared cultures were counted. Typical passage infections used 1.6 × 10^6 ^cells per well in a six-well plate. For fixed-MOI cultures the virus level from each passage was determined by plaque assay and diluted using cell culture media with 2% FBS to set the MOI for the next passage. To infect, 100 microliters of the diluted virus solution was added to the cell monolayer. The dish was then swirled so that the virus solution would fully coat the cell monolayer. After incubation at 37°C for 1 h, the virus solution was removed, and 2 ml of cell culture media with 2% FBS was pipetted into the well. The wells were then incubated at 37°C for 23 h. After this incubation period the solution was mixed by pipetting, aliquoted, and stored at -90°C until diluted for titering or use in a subsequent infection. For fixed-volume passages, cell culture media containing 2% FBS was added to the well immediately upon removal of the media containing 10% FBS. Each passage infection was initiated by transferring 20 microliters of the mixed virus solution from the previous passage. The infection was then incubated at 37°C for 24 hours and harvested, as described above.

### Model construction

As detailed in the Results section, difference equations linking inputs and outputs of infection were employed to estimate overall yields of virus and DI particles. Parameter values (p) were estimated by least squares:

$$\underset{p}{\min}{\sum \left[\log_{10}(observed)-\log_{10}\left(predicted\right)\right]}^{2}$$

with the summation spanning the available data points. The corresponding confidence intervals were constructed by parametric bootstrapping percentile method using multiple resamples from the fitted model as detailed elsewhere [31].

## Results

### Fixed-volume versus fixed-MOI passaging

**Figure 1:**
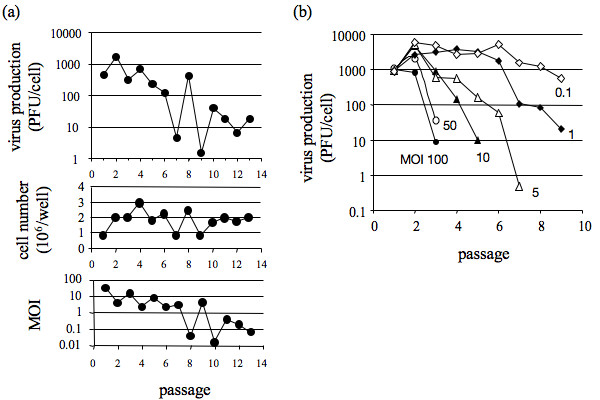
**Virus production during passage cultures**. (a) Virus production during fixed-volume passages (upper panel), with corresponding cell number (middle panel) and multiplicity of infection (MOI, lower panel); (b) Virus production during fixed-MOI passages for MOI of 100 (filled circles), 50 (open circles), 10 (filled triangles), 5 (open triangles), 1 (filled diamonds), and 0.1 (open diamonds); cultures were stopped when virus yields fell below levels needed to maintain the MOI.

Over the course of 13 fixed-volume passages levels of virus production from cell monolayers dropped more than 10-fold, and passage-to-passage titers of virus fluctuated up to 100-fold (Fig. 1a), producing drops and recoveries in virus yield that are indicative of infections containing mixtures of virus and its defective interfering particles [5,32]. These changes in virus yield during fixed-volume passaging also impacted the passage-to-passage MOI, which varied from above 10 to below 0.1 (Fig. 1a). When passages were performed with fixed MOI, rather than fixed volume, more regular and rapid declines in virus productivity occurred at higher MOI passages (Fig. 1b), providing evidence that DI particles accumulate in populations more rapidly during passage at high MOI. For passages at MOI 100 and 50, the average virus yield dropped 10-to-100 fold by passage three, a loss that required nine passages at MOI 1.

**Figure 2:**
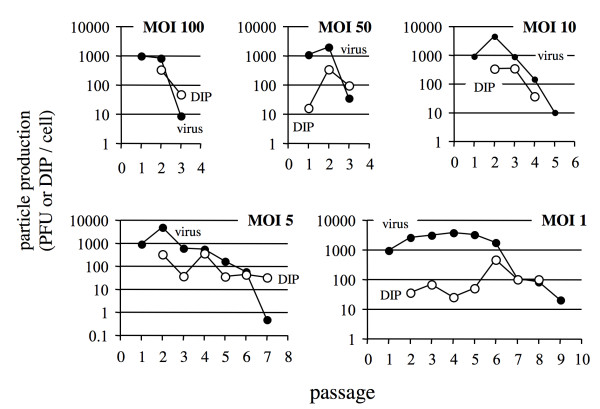
**Productivity of virus and DI particles during fixed -MOI passage**. Passages were carried out at MOI 100, 50, 10, 5 and 1. Closed symbols are virus yield (PFU/cell) and open symbols are DI particle yields (DIP/cell).

To better understand how the accumulation of DI particles across fixed-MOI passages impacts virus yields we quantified the activity of interfering particles in different passage populations using the yield-reduction assay (Fig. 2). At high MOI (100 and 50) a large drop in virus levels between passages 2 and 3 was accompanied by a significant DI particle activity by passage 2. In both cases DI particle activity surpassed virus activity by passage 3. Passaging at MOI 10 brought about a more gradual decline of virus activity than passaging at higher MOIs, and DI particle levels did not surpass levels of infectious virus. For the lowest MOIs (5 and 1), emerging DI particles fluctuated in their activities, and more passages were required to cause the largest drops in virus yield. In most cases DI particle production in passage 1 was below the detection limit (see the exception for MOI 50), so data points were not shown.

### Model of virus and DI particle population dynamics

To quantitatively assess the effects of virus-DI particle interactions during serial-passage culture we applied a mathematical model that we recently developed to quantitatively account for the input-output behavior of single passages [28]. Briefly, the model accounts for cells (C), virus particles (V), and interference equivalents (IE). An IE is defined experimentally as the quantity of DI particles that fully inhibit production of viable virus particles when delivered to an infected cell. Historically, it was assumed that the presence of single DI particle during co-infection with virus could fully inhibit production of viable virus particles [30]. However, our recent study suggests this classical assumption has limitations [28]. In our model we assume that each DI particle contributes an equivalent share of the total interference, a quantity we define as one interference unit (IU). It follows that multiple IUs may be need to achieve the effect of one IE; in our model the parameter *z *is the number of IUs per IE. The model equations are:

(1) $$V_{N+1}=C_{V}\cdot b+C_{B}\cdot \left(b-p\cdot \log\left(\frac{IE_{N}}{C_{N}}\cdot z\right)+q\cdot \left(\frac{IE_{N}}{C_{N}}\right)\right)$$

(2) IEN+1=CB⋅ (d+r⋅log(IENCN⋅z)−s⋅log[(IENCN⋅z)−w]+)+g⋅CV

(3) $$C_{V}=e^{\left(-\frac{IE_{N}}{C_{N}}\cdot z\right)}\cdot \left(1-e^{\left(-\frac{V_{N}}{C_{N}}\right)}\right)\cdot C_{N}$$

(4) $$C_{B}=\left(1-e^{\left(-\frac{IE_{N}}{C_{N}}\cdot z\right)}\right)\cdot \left(1-e^{\left(-\frac{V_{N}}{C_{N}}\right)}\right)\cdot C_{N}$$

where the output concentration of virus particles from the N^th ^passage (V_N+1_) depends on virus amplification in cells initially infected with either virus alone (C_V_) or cells co-infected with both virus and IEs (C_B_). The output concentration of IEs from the N^th ^passage (IE_N+1_) depends on their average production from co-infected cells (C_B_) and their generation from IE-free infected cells (C_V_). To determine how the initial number of cells (C_N_) distribute among those that contain virus alone (C_V_) or virus and IEs (C_B_) we assume that particles encounter and infect cells following a Poisson distribution, as shown in equations 3 and 4. The model has nine parameters, presented in Table 1. In addition to *z*, three parameters describe the productivity of virus, and five parameters describe the productivity of IEs. In the absence of DI particles a virus-infected cell produces an average of *b *virus particles. This productivity level is then modified by parameters *p *and *q *which describe how the virus productivity changes depending on the dose of interference. The negative sign on *p *reflects the reduction in virus yield from DI particle co-infection, and the positive value of *q *accounts for a partial recovery of virus yield for large co-infecting doses of DI particles. The IEs are either generated *de novo *from virus-infected cells (C_V_), described by the parameter *g*, or amplified from co-infected cells (C_B_), described by the parameter *d*. The effects of IE co-infection on IE yield is then modified by the parameters *r*, *s*, and *w*. At low levels IEs enhance their own production, reflected by the positive value of *r*. At higher IE, where *(IE_N·_z/C_N_) *is greater than *w*, then IEs inhibit their own production, reflected by the negative value on *s*. The notation ( )_+ _indicates that only the positive part of the difference is used, so the *s*-term only contributes to IE when *(IE_N·_z/C_N_) *exceeds *w*. These parameters were estimated from the single-passage productivity of both virus and IEs, quantified from cells co-infected by serial dilutions an IE-rich inoculum and excess virus [28].

**Table 1:** Model parameter values.

Parameter Description		Value	95%	Units
Wild type virus burst size	b	2670	± 160	PFU/cell

IE burst size	d	420	± 10	IE/cell

Factor to describe an IEs as multiple particles	z	190	± 10	IU/IE

Extent of yield decrease of wild type virus with additional IUs present	p	880	± 20	$\frac{PFU}{cell\cdot \log\;\left(\frac{IU}{cell}\right)}$

Extent of yield increase of wild type virus at very high interference	q	0.47	± 0.10	PFU/IU

Cut-off between high and very high interference	w	290	± 150	IU/cell

Extent of yield increase of IE with additional IUs present	r	30	± 5	$\frac{IE}{cell\cdot \log\;\left(\frac{IU}{cell}\right)}$

Extent of yield decrease of IE with additional IUs present above w	s	80	± 5	$\frac{IE}{cell\cdot \log\;\left(\frac{IU}{cell}\right)}$

IE generation rate	g	0.001	± 0.0002	IE/cell

**Figure 3:**
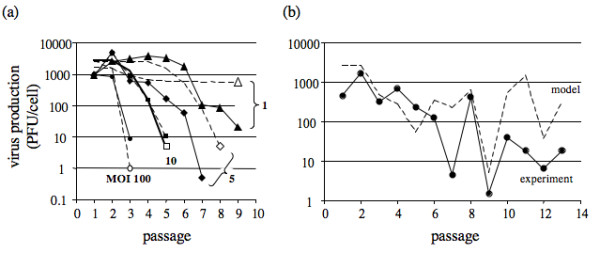
**Comparison of experimental data with models**. (a) Fixed-MOI passages, with data (filled symbols, solid lines) and model (open symbols, dashed lines) and (b) fixed-volume passages. The data are from Fig. 1, and predictions from the model incorporate experimentally measured cell levels at each passage. The initial virus concentration (passage 1) for models is based on the measured stock virus titer, where we assume the stock virus was free of any DI particles or IE activity. Models all used parameters from Table 1.

For fixed-MOI passages the model captured several essential features of the data (Fig. 3a): a more than 100-fold drop in virus production between passages 2 and 3 at MOI 100, trailed by a two-passage lag for drops in virus production at MOI 10, and a further lag for MOI 5. At MOI 5 the model matched the overall trend of the observed decline in virus production but it overestimated virus productivities at each passage by 4- to 150-fold. Predictions for MOI 1 showed the greatest deviations, with observed titers dropping 150-fold over 9 passages while predicted trajectories stayed level. For fixed-volume passages the model captured overall observed declines in virus productivity as well as major fluctuations between passages 6 and 9, as shown in Fig. 3b. Greater deviation between the model and the data appeared for later passages, where the model predicted greater recovery of virus production than observed for passages 10 to 14.

### Sensitivity of simulated fixed-volume passages to inputs

Our model for fixed-volume passages depends on the volume of sample transferred at each passage, the number of cells infected, and levels of virus and IEs in the initial culture. Differences in initial levels of virus had negligible effects on overall yields, so we used our model to test how variation in other the transfer volume and cell number affected the simulated or predicted population behavior.

**Figure 4:**
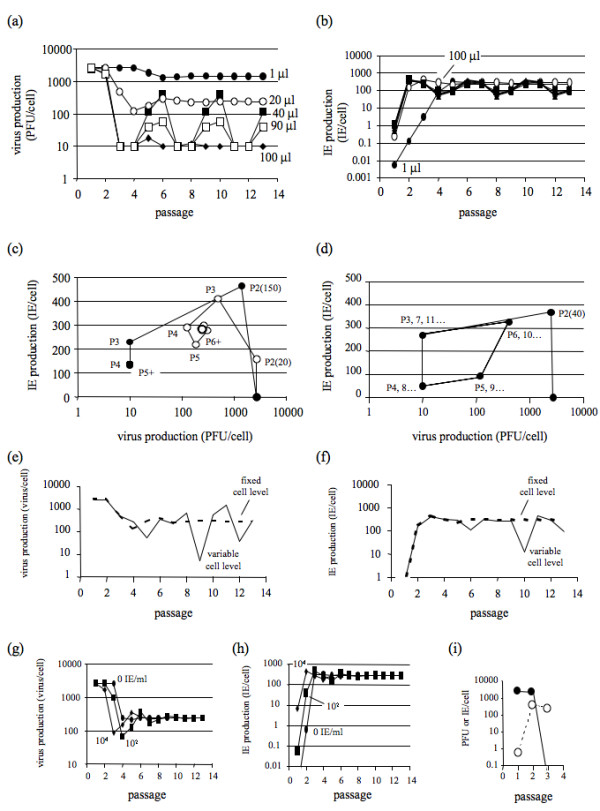
**Sensitivity of model predictions to different inputs**. Effects of passage volume on (a) production of virus, and (b) production of interference equivalents (IE). Trajectory plots for (c) virus and IE production for 20- and 150-microliter passages, and (d) virus and IE production for 40-microliter passages. Predicted effects of cell level on (e) virus production and (f) IE production using the experimentally observed cell levels (solid lines), and constant cell number of 2 × 10^6 ^cell/passage (dashed lines). Effect of initial IE concentration on (g) virus production and (h) IE production. (i) Conditions for curing of infection for virus (filled circles) and IE (open circles); here the value of parameter q was set to zero and the simulation was implemented for fixed-volume passages with 50 microliters. Simulation input values for passage 1 were 1 × 10^9 ^PFU/ml, 400 IE/ml, 2 × 10^6 ^cell/passage, and 2-microliter passaged volumes from total culture well (2 ml), unless otherwise noted.

When transfer volumes were increased from 1 to 100 microliters simulated virus production levels dropped and IE activities rose rapidly (Fig. 4a/4b), reflecting the dominating effects of transferring more IEs from one passage to the next. For transfer volumes of 20 microliters or less, a steady-state arose from a balance between effects of IE depletion by dilution and IE replenishment by generation and replication.

Oscillations in virus and IEs occurred at intermediate transfer volumes, as shown for 40 and 90 microliter transfers. At transfer volumes of 100 microliters IE production dominated, and low productivities and levels of virus particles served to sustain the IE population.

The interactions between virus and IE levels across passages may be summarized by plotting the IE productivity versus virus productivity at each passage, showing both steady-state (Fig. 4c) and oscillatory (Fig. 4d) behaviors. Trajectories start from the x-axis where the initial virus levels are high and IE levels are low. From passage 1 to passage 2 the IE yield increases with little reduction in the virus productivity. For relatively low transfer volumes of 20 microliters the trajectory coils counterclockwise as it approaches the steady-state (Fig. 4c). The coil shape is indicative of damped oscillations in both the virus and IE yield during passaging. For larger transfer volumes, cultures span greater ranges in productivities, as shown for a transfer volume of 40 microliters (Fig. 4d), where trajectories stably oscillate without dampening to a steady state. As the transfer volume increases from 40 to 90 microliters the amplitude of the oscillations decreases (Fig. 4a). As transfer volumes increase still further amplitudes of oscillation (and magnitude of trajectory loops) decrease until the oscillations cease at transfer volumes of at least 100 microliters, as shown in Fig. 4a/4c. Under such conditions, the trajectory reaches a maximum IE productivity at passage 2, both yield of virus and IE drops by passage 3, and the virus yield continues to drop until the steady state is reached.

The impact of host-cell availability for infection was tested by simulating passages using either fixed or fluctuating cell numbers. Fluctuating conditions were simulated by using cell numbers that were experimentally measured during the fixed-volume passage, at the time of infection (Fig. 1a). Both virus and IE productivity exhibited 10-100 fold changes in passage-to-passage levels, as shown in Fig. 4e and Fig. 4f, respectively. The most distinguishing feature was a major drop in productivity of wild-type virus and IEs in passages 9 and 10, respectively. Passage 9 had a relatively low cell count at 0.83 × 10^6 ^per well, where the mean across all passages was (1.8 ± 0.6) × 10^6 ^cells per well, respectively.

The model was used to simulate the effects of different initial IE levels on the behavior of virus and IE levels across fixed-volume passages, as shown in Fig. 4g and Fig. 4h, respectively. Initial levels from zero to 10^4 ^IE per ml had no effect on the productivity of virus or IE at steady state. However, transients in virus and IE productivity differed in duration, requiring up to 9 passages to achieve steady-state levels.

### Curing of infection

Finally, we used the model to explore conditions for spontaneous curing of infection, where the production of virus can be driven to zero. Fig. 4i shows an example of a spontaneously cured infection. For the infection to spontaneously cure, the model requires that co-infections produce no virus. This condition was implemented in the model by setting *q *= 0, rather than *q *= 0.47 (Table 1).

## Discussion

By maintaining a fixed multiplicity of infection (MOI) across serial virus passages we observed a regular pattern of population behavior: average yields of virus declined more rapidly for passages performed at higher MOIs (Fig. 1b). The earlier and faster decline in virus level at higher MOI's could be attributed most simply to smaller dilution effects. At higher MOI more virus and more DI particles are transferred from one generation to the next, enabling more rapid accumulation of DI particles and greater inhibitory effects on virus growth. Alternatively, high MOI infections in the absence of DI particles could be associated with intrinsically higher rates of *de novo *DI particle production. To test this possibility we allowed for *de novo *DI particle generation in our model and found that declines in virus yields for passages at MOI 100 and MOI 10 could be reasonably accounted for by assuming a fixed intrinsic rate of *de novo *DI particle generation (g = 0.01 IE/cell), as shown in Fig. 3a. In contrast, the model failed to capture the extent of observed yield reduction for passages performed at MOI 5 and MOI 1. For these lower MOI a better agreement between the model and the data could be obtained by incorporating a significantly higher rate of DI particle generation into the model (g = 20 IE/cell, not shown). A higher DI particle generation rate for conditions of lower MOI may initially seem paradoxical, because the generation of DI particles should depend on the number of replication events, which would be reduced at lower MOI. However, it should be noted that the DI particle generation rates are expressed as IE generated per *infected *cell. Other mechanisms may also play a role in the mismatch between model and data at low MOI. For example, DI particle populations may evolve to more potently inhibit amplification of virus in cells co-infected with both virus and DI particles, or *de novo *generated DI particles may emerge and displace existing DI particles [18,33,34]. Further studies on isolated DI particle sub-populations will be needed to elucidate mechanisms responsible for the differences between the model and data at low MOI.

What causes the chaotic population dynamics that is a hallmark of fixed-volume serial virus cultures? Mathematical models of passage behavior have previously suggested how chaotic behavior can arise during fixed-volume passages [21], but these models have been based on assumptions that cells co-infected with standard and DI particles produce only DI particles. They have also assumed constant yields of DI particles are produced from co-infected cells. These assumptions are not generally valid based on our recent experimental and modeling studies of VSV growth on BHK cells [28]. Specifically, co-infected cells produce both virus and DI particles rather than only DI particles. Moreover, average yields of virus and DI particles determined from co-infected cells were not constant; instead, they depended on DI particle dose. These experimental results were used here to develop new mathematical models and simulate the behavior of virus and DI particle populations in fixed-volume passages. The simulated populations exhibited no chaotic behavior. Instead, initial transients spanning up to six passages settled into either steady-state or regular oscillatory behaviors (Fig. 4a-d). Experimental measures of standard and DI particle behavior over fixed-volume VSV passages have shown cyclic behavior [32], but the observed six passages were too few to establish whether regular oscillations were feasible. We carried out fixed-volume passages in the laboratory and observed irregular changes in the virus productivity from one passage to the next, most notably between passages 6 and 10 (Fig. 1a). When we expanded the model to incorporate experimentally measured host-cell levels at each passage then the predicted virus levels exhibited significant fluctuations, especially beyond passage 6, with a greater than 100-fold drop between passages 8 and 9, as observed in experiments (Fig. 3b and Fig. 4e/4f). Relatively small changes in cell levels can give rise to large changes in virus production from infected and co-infected cells, owing to the non-linear relationship between virus production(*V_N+1_*) and cell level(*C_N_*) shown in Eq. 1. A testable prediction of the model is that by controlling experiments to reduce fluctuations in cell levels one should observe more regular behavior of virus levels (e.g., steady-state or regular oscillations) during constant-volume passages, as reflected in Fig. 4a-d. More broadly, these results show that passage-to-passage changes in the virus level can arise from competition between virus and DI particles for host-cell resources, as well as from fluctuations in the level of host cells. From an ecological perspective, fluctuations in host cells may be viewed as periodic mortality events that can significantly impact the predator-prey dynamics [35].

To run our model one must provide an initial condition that specifies not only the concentration of virus particles, but also the concentration of DI particles in the initial passage. In the laboratory, the concentration of DI particles in the initial passage will depend on the history of the stock solution. For example, if the stock is amplified from an isolated plaque at low MOI or serially passaged at high MOI it may contain negligible or high levels of DI particles, respectively. In our model this input was found to have no effect on the steady-state dynamics of the virus and DI particle populations (Fig. 4g/4h). Even in the absence of initial DI particles the *de novo *generation of DI particles produced interference activity that was rapidly amplified in co-infections with wild-type virus. It appears that once DI particles are available they will tend to dominate subsequent co-infections. While new or different DI particles could, in principle, be generated by *de novo *processes, the low productivity of such processes prevents *de novo *DI particles from being competitive with existing DI particles. This dominance of initial DI particles has been found experimentally by observing how parallel cultures initiated from a common stock preserve DI particles present in the stock [36].

Some cells can make nearly a million-fold more DI particles than other cells, depending on how they are infected. When a cell is infected with viable virus, in the absence of any DI particles, few if any DI particles will be made, reflecting the small *de novo *rate of DI particle generation. This rate was estimated for VSV at 0.001 IE/cell. However, when a cell is co-infected with viable virus and DI particles, then many DI particles can be made, reflecting their ability to divert resources from virus to DI particle production.

Experiments suggest that more than 500 IE per cell can be produced under optimal conditions for DI particle production [28]. If we assume a single DI particle corresponds to a single interference unit (IU), then we may estimate that optimal condition for DI particle production make (500 IE/cell × 200 IU/IE × 1 DI particle/IU) or 100,000 DI particles per cell. At the other end of the spectrum, the least productive or *de novo *rate of DI particle generation is (0.001 IE/cell × 200 IU/IE × 1 DI particle/IU) or 1 DI particle from every five infected cells. Thus, a 500,000-fold difference in DI particle yields separates the most productive from the least productive cells. A more expanded view of productivity, which includes the production of both DI and viable particles, may be concisely expressed in terms of a particle-to-PFU ratio. Such a ratio can be experimentally determined by estimating the number of virus-sized particles in a sample by electron microscopy and dividing by the number of viable virus particles measured by the plaque assay. If we assume that only DI and viable particles are produced by a co-infected cell, then we estimate a particle-to-PFU ratio of 38 (or 102,670 particles/2670 PFU) based on maximal production of DI and viable particles. This estimate is consistent with the observed range for VSV from 10 to 50 particle-to-PFU [37].

Although spontaneous curing of virus infections by DI particles has not been experimentally observed, the possibility has been explored in previous models [21,22]. Our data-driven model indicates VSV infections cannot be spontaneously cured because infected cells produce viable virus under all conditions, even when DI particles most potently interfere with virus production. If desired, our model could be adapted to simulate a curing effect by setting the parameter *q *to zero, so cells co-infected with virus and a high level of DI particles would produce only DI particles. Others have found that individual cells isolated from a co-infected cell population produced no detectable virus, suggesting a curing effect, but other cells drawn from the same population did produce virus [38]. The absence of experimental support to date for spontaneous curing of infections by DI particles should not detract from advancing their applications. DI particles have potentially therapeutic applications as enabling tools for vaccination [39,40], as prophylactics, or as therapeutics [41]. In an infected patient, we expect that virus and DI particles would grow and spread continuously on susceptible host cells, so our current model would have limited application owing to its focus on discrete population passages. Our data-driven modeling approach could, however, be extended to develop a continuous rather than discrete model. We envision that such a data-driven continuous model would be based on experiments of growth kinetics that explicitly accounted for the dynamic interactions between cells, virus and DI particles.

We are seeking to develop experimental measures and theoretical methods to better understand how viruses grow and how their infections spread. Our main contribution here has been to better link quantitative experiments of virus and DI particle populations with quantitative models. This approach will be enhanced in the future by incorporating additional measures, beyond plaque counting, including electron microscopy (particle counting), quantitative PCR, and next-generation sequencing. Ultimately, our approach aims to provide a predictive capability that will enable the long-term control of virus infections.

## Conclusions

Serial-passage cultures of virus at fixed multiplicity of infection (MOI) produced regular patterns of attenuated virus production across passages. These patterns of steeper declines in virus production at higher MOI could be accounted for by a mathematical model that incorporated interactions among virus particles, defective interfering (DI) particles, and cells to predict yields of virus and DI particle production from co-infected cells. Further, analysis of fluctuating virus populations provided evidence for a high sensitivity of virus and DI particle population dynamics to small changes in the levels of cellular resources.

## Competing interests

The authors declare that they have no competing interests.

## Authors' contributions

KT and JY conceived of the study, and KT performed all experiments. KT and JY analyzed the data and wrote the manuscript.
